# Carboxylate-Terminated Electrode Surfaces Improve the Performance of Electrochemical Aptamer-Based Sensors

**DOI:** 10.1021/acsami.4c21790

**Published:** 2025-01-22

**Authors:** Rose Mery Bakestani, Yuyang Wu, Bettina Glahn-Martínez, Tod E. Kippin, Kevin W. Plaxco, Ruben W. Kolkman

**Affiliations:** † Department of Chemistry and Biochemistry, University of California Santa Barbara, Santa Barbara, California 93106, United States; ‡ Department of Analytical Chemistry, Faculty of Chemistry, Universidad Complutense de Madrid, 28040 Madrid, Spain; § Department of Psychological and Brain Sciences, University of California Santa Barbara, Santa Barbara, California 93106, United States; ∥ Biological Engineering Graduate Program, University of California Santa Barbara, Santa Barbara, California 93106, United States

**Keywords:** electrochemical aptamer-based sensors, electrochemical biosensing, self-assembled monolayer, surface chemistry, square-wave voltammetry

## Abstract

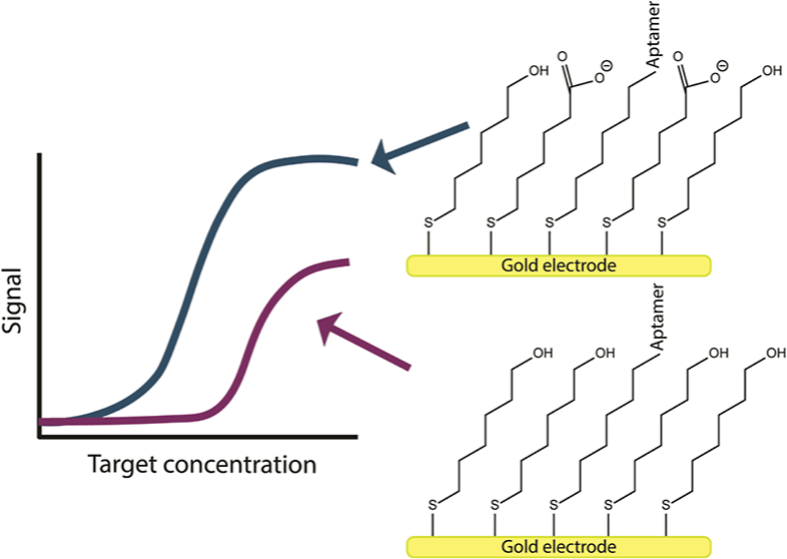

Electrochemical aptamer-based (EAB) sensors are a molecular measurement platform that enables the continuous, real-time measurement of a wide range of drugs and biomarkers in situ in the living body. EAB sensors are fabricated by depositing a thiol-modified, target-binding aptamer on the surface of a gold electrode, followed by backfilling with an alkanethiol to form a self-assembled monolayer. And while the majority of previously described EAB sensors have employed hydroxyl-terminated monolayers, a handful of studies have shown that altering the monolayer headgroup can strongly affect sensor performance. Here, using 4 different EAB sensors, we show that the mixed monolayers composed of mixtures of 6-carbon hydroxyl-terminated thiols and varying amounts of either 6- or 8-carbon, carboxylate-terminated thiols lead to improved EAB sensor performance. Specifically, the use of such mixed monolayers enhances the signal gain (the relative change in the signal seen upon target addition) for all tested sensors, often by several fold, both in buffer and whole blood at room temperature or physiological temperatures. Moreover, these improvements in gain are achieved without significant changes in the aptamer affinity or the stability of the resulting sensors. In addition to proving a ready means of improving EAB sensor performance, these results suggest that exploration of the chemistry of the electrode surface employed in such sensors could prove to be a fruitful means of advancing this unique in vivo sensing technology.

## Introduction

The ability to measure the concentrations of specific molecules in unprocessed bodily fluids has the potential to significantly impact healthcare by providing a real-time window into molecular health status and improving the precision and convenience with which pharmacological treatments are delivered and their effects monitored.^1,2^ The convenient, real-time measurement of blood sugar, for example, has greatly advanced the treatment of diabetes.^3,4^ The technology underlying the widely used glucose sensor, however, employs the enzymatic conversion of the target molecule into an easily detectable product, an approach that, unfortunately, is challenging, if not impossible, to translate to the detection of most molecular targets.^5−8^ In response, we^9,10^ and others^11−13^ are developing electrochemical aptamer-based (EAB) sensors, a class of biosensors that, while relying on a biomolecular affinity reagent to recognize its target, does not depend on any chemical transformation of the target, rendering the approach quite general. For example, to date, EAB sensors have been shown to support the continuous measurement of a wide range of metabolites,^14,15^ toxins,^16,17^ therapeutic drugs,^18,19^ and psychostimulants,^20−22^ in real time, and in complex sample matrices, including in situ in the body in the blood (plasma), brain (cerebrospinal spinal fluid), and solid peripheral tissues (interstitial fluid).^23−26^

**Figure 1:**
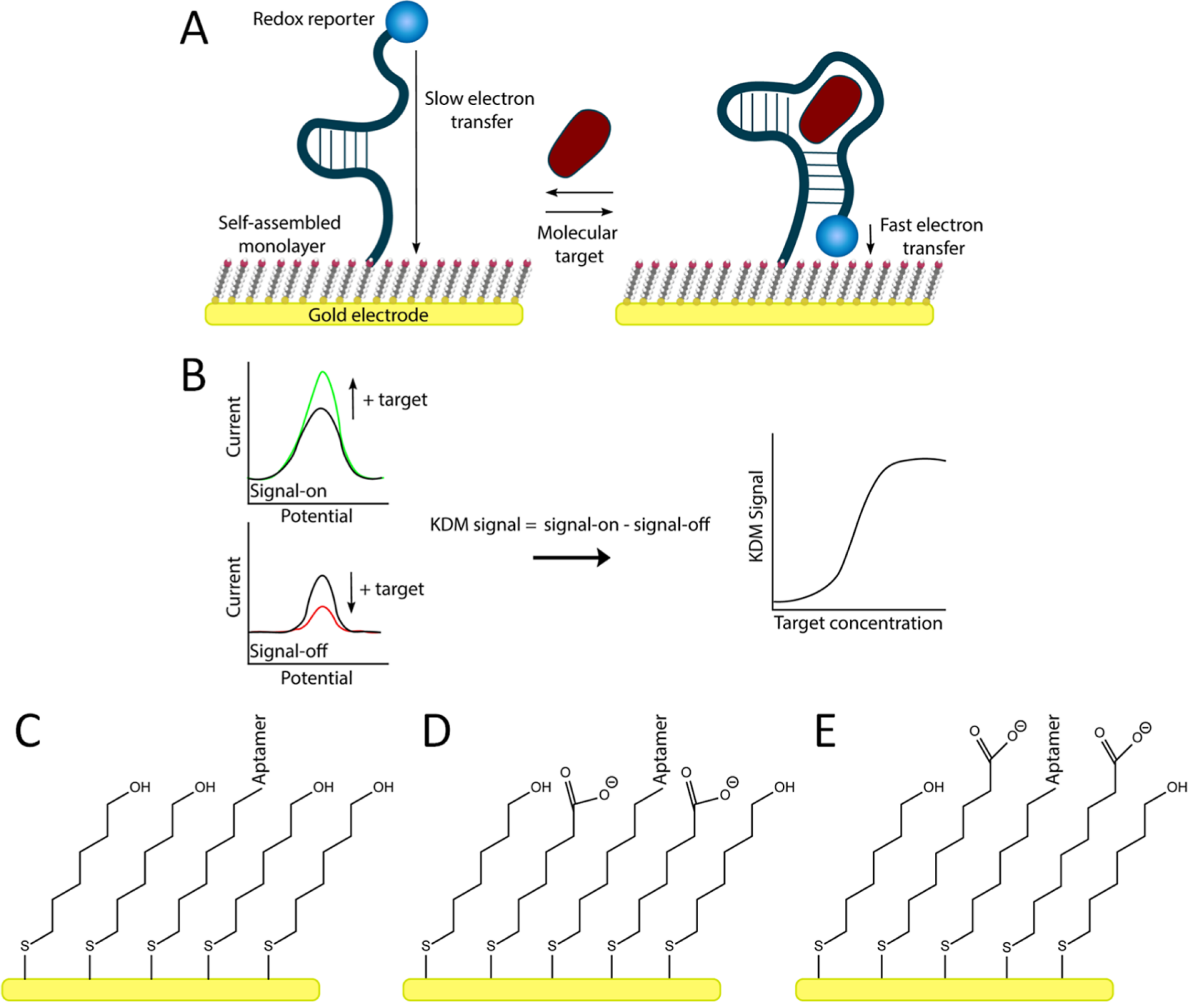
(A) EAB sensors are composed of a redox-reporter-modified aptamer attached to a gold electrode via an alkanethiol SAM. The aptamer is engineered such that it undergoes a binding-induced conformational change, altering the position of the reporter and producing (B) an easily measurable change in the electrochemical signal. Specifically, we monitor target binding using SWV which, depending on the square-wave frequency employed, responds in either a “signal-on” or a “signal-off” manner.^28^ To correct for the drift seen when sensors are deployed in 37 °C, bodily fluids, we employ KDM, which takes the difference between the signals seen at signal-on and signal-off square-wave frequencies.^14,36,37^ (C) In most prior studies of EAB sensors, the alkanethiol SAM is composed of 6-mercaptohexanol, producing a hydroxyl-terminated surface.^29^ Here, in contrast, we explore the performance of EAB sensors fabricated using mixed monolayers composed of 6-mercaptohexanol and either a (D) 6-carbon carboxylate or (E) 8-carbon carboxylate.

EAB sensors are composed of a target-recognizing, redox-reporter-functionalized aptamer attached to an interrogating gold electrode via an alkanethiol self-assembled monolayer (SAM) (Figure 1A), which, when interrogated using square-wave voltammetry (SWV), produces an easily measurable output signal (Figure 1B).^27,28^ The large majority of previously reported EAB sensors employed 6-mercaptohexanol to form this SAM, producing a hydroxyl-terminated surface^29^ (Figure 1C) that provides good stability, relatively rapid electron transfer rates, and acceptable antifouling properties.^30^ This said, a number of researchers have explored how the properties of the SAM alter EAB sensor performance, finding that EAB signaling is sensitive to SAM thickness,^31^ to the density with which the aptamers are packed on the SAM surface,^32^ and to the chemistry of the SAM headgroup.^29,31,33^ Sulfonate and amine headgroups, for example, have been shown to increase the signal gain (the relative change in signal upon the addition of a saturating target) of EAB sensors but often at the cost of poor reversibility.^31^ Similarly, the use of SAMs composed of methyl headgroups improves sensor stability but often at the cost of decreased aptamer affinity (*K*_d_) and, with that, poorer limits of detection (LOD).^29^ Finally, the use of zwitterionic headgroups, such as phosphatidyl choline, has been shown to reduce the drift seen when EAB sensors are deployed in vivo, but this likewise occurs at the cost of poorer LOD and an inability to correct any residual drift using kinetic differential measurements (KDM, Figure 1B), a dual-frequency SWV technique often used to enable sensor deployment in vivo.^34,35^ It thus appears that, at best, most prior efforts to change the SAM headgroup employed in EAB sensors improved one aspect of sensor performance while harming another. In contrast, however, Kesler et al.^33^ recently reported that the use of a homogeneously carboxylate-terminated SAM simultaneously increases both the gain of a doxorubicin-detecting sensor and its target affinity. Building on these prior observations, here, we explore the impact of both homogeneously carboxylate-terminated SAMs and mixed carboxylate-/hydroxyl-terminated SAMs (Figure 1D,E) on the performance of EAB sensors under clinically realistic testing conditions.

## Results and Discussion

As our exploration test beds, we employed 4 previously reported EAB sensors: 1 against the targets tryptophan,^38^ 2 against the target vancomycin, with one of the latter employing a 45-base aptamer (referred to as vanc-45)^18^ and the second employing a completely distinct (i.e., no significant sequence similarity) 28-base aptamer (referred to as vanc-28) and, in some studies, a sensor against the anesthetic procaine.^23^ Following traditional EAB sensor fabrication methods,^14,39^ we modified each aptamer with a 6-carbon thiol and the redox reporter methylene blue. After depositing the thiolated aptamer on a gold electrode, we formed the requisite SAM by backfilling with (i) pure 6-mercaptohexanol, (ii) pure 6-mercaptohexanoic acid, (iii) various stoichiometric ratios of 6-mercaptohexanol and 6-mercaptohexanoic acid (Figure 1D), or (iv) various stoichiometric ratios of 6-mercaptohexanol and 8-mercaptooctanoic acid (Figure 1E). Of note, previous studies suggest that the carboxylate content in such a mixed SAM will depend monotonically on the stoichiometric ratio of the thiols employed during the deposition step.^40−42^ Following fabrication, we interrogated the resulting sensors using SWV performed at a pair of “signal-on” and “signal-off” frequencies, as required to perform KDM (Figure 1B), an approach that increases EAB signal gain and corrects the drift inevitably seen in vivo.^36,37^

**Figure 2:**
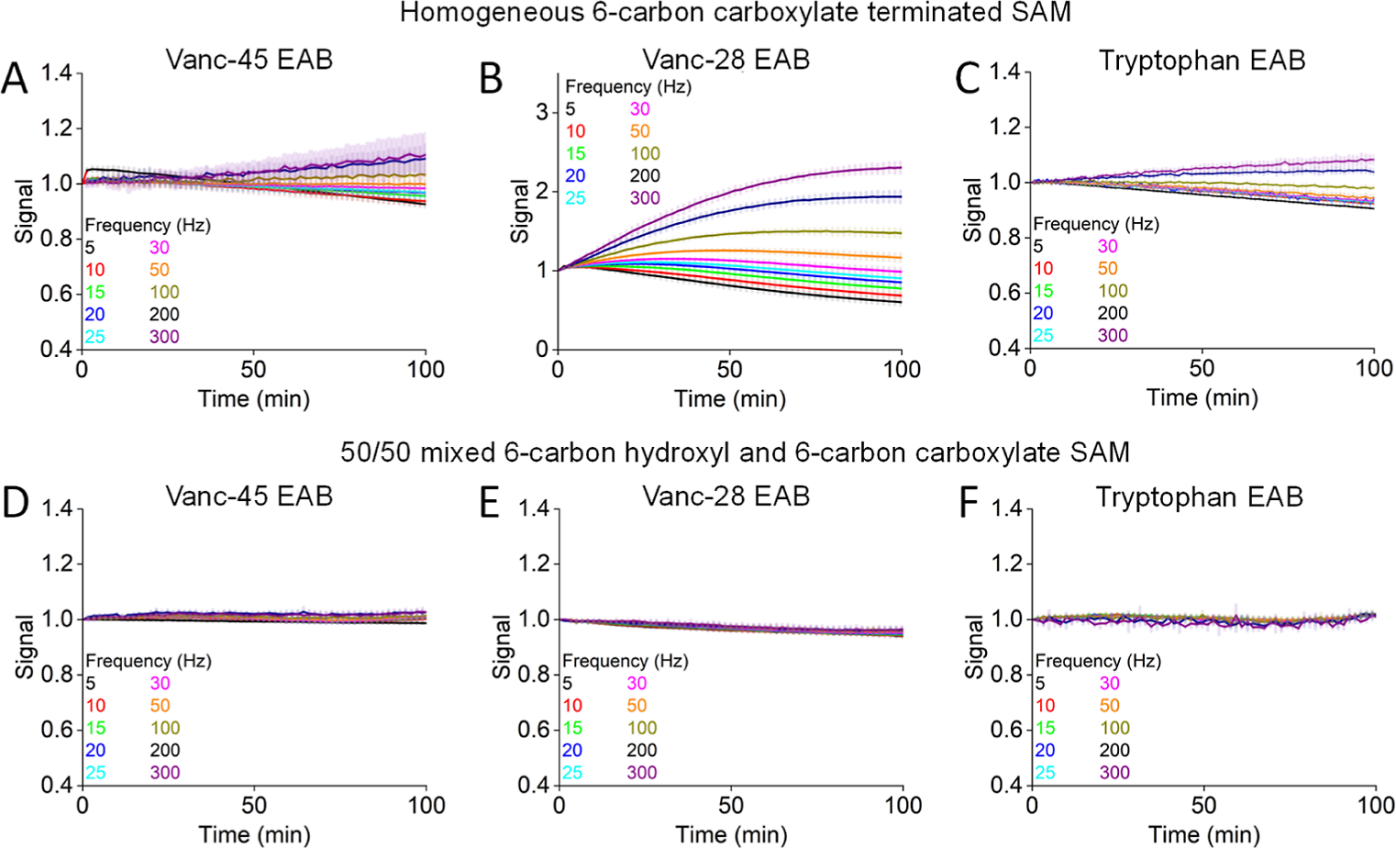
(A–C) Even when challenged in simple room-temperature PBS are EAB sensors fabricated using homogeneously 6-carbon-carboxylate-terminated SAMs relatively unstable. (D–F) In contrast, sensors fabricated using 1:1 mixtures of 6-carbon carboxylate- and 6-carbon hydroxyl-terminated thiols are quite stable. Shaded areas indicate in this and the following figures the standard deviation from at least three independently fabricated electrodes. Note: the sequences of vanc-45 and vanc-28 exhibit no significant similarity, and thus, these are 3 completely unrelated sensors.

Although Kesler et al.^33^ have reported that the use of a homogeneously carboxylate-terminated SAM improved the performance of a doxorubicin-detecting sensor, here, we find that the stability of sensors fabricated from such SAMs is rather poor. To show this, we fabricated sensors using only a 6-carbon carboxylate-terminated thiol and monitored their square-wave peak currents when interrogated in room-temperature phosphate buffered saline (PBS). The resulting signals were not stable at any of the square-wave frequencies that we employed for any of the three sensors (Figure 2A–C). Given the relatively poor stability of these homogeneously 6-carbon carboxylate-terminated SAMs, we did not explore them further in our work. Instead, we next characterized sensors employing a 50% 6-carbon carboxylate/50% 6-carbon hydroxyl headgroup mixed SAM, under the argument that, by reducing repulsion between neighboring headgroups, this would lead to more stable SAMs and thus more stable sensors. Consistent with this, we find that sensors employing these mixed monolayers are quite stable (Figure 2D–F).

**Figure 3:**
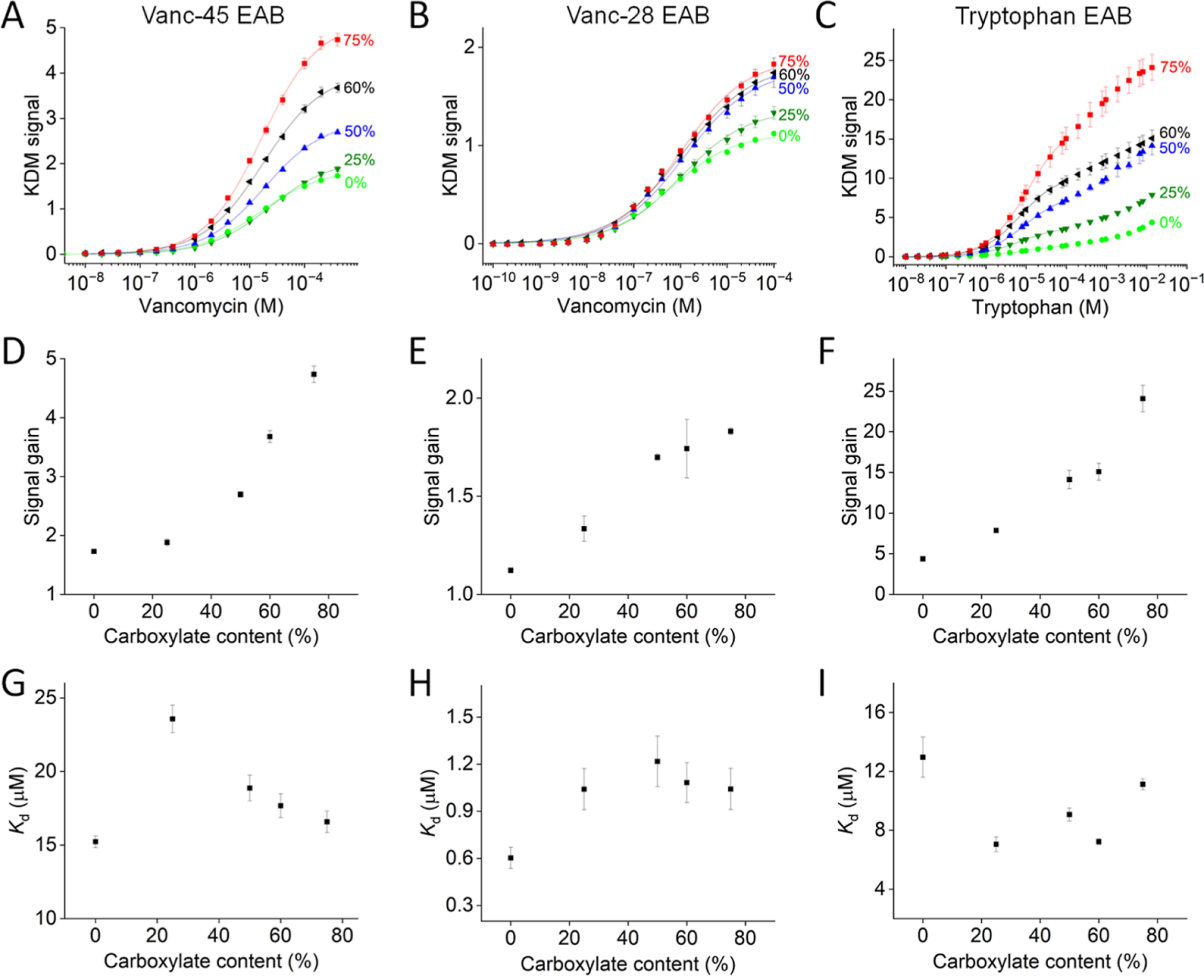
When tested in PBS at room temperature, the signal gain of EAB sensors improves upon the introduction of increasing substoichiometric levels of 6-carbon carboxylate in the SAM. Shown are titrations of (A) vanc-48, (B) vanc-28, and (C) tryptophan sensors fabricated using varying fractions of 6-carbon carboxylate-terminated thiols in the SAM. Note that the tryptophan sensor exhibits biphasic binding,^38^ which becomes more prominent as the affinities of the two binding events diverge at lower carboxylate content. The increase in (D–F) signal gain is monotonic with increasing 6-carbon carboxylate levels. Here, we defined signal gain as the relative signal change between 0 and 400 μM vancomycin (vanc-48), 0 and 100 μM vancomycin (vanc-28), and 0 and 14 mM tryptophan. (G–I) *K*_d_ changes as a function of the 6-carbon carboxylate content. The reported *K*_d_ of the tryptophan sensors is that of the higher affinity binding event. Error bars in this and the following figures indicate the standard deviation of four independently fabricated electrodes.

The use of mixed, 6-carbon carboxylate-terminated and 6-carbon hydroxyl-terminated SAMs improves EAB sensor gain (Figure 3 and Table S1). To show this, we challenged sensors in room-temperature PBS with stepwise increasing target concentrations. Under these conditions, vanc-45 sensors employing 6-carbon hydroxyl SAMs exhibit the characteristic Langmuir isotherm binding curve with a *K*_d_ of 15.2 ± 0.4 μM (all “error bars” denote the standard deviation of four independently fabricated electrodes) and a gain of 173 ± 2% (at 400 μM target concentration). When the same sensor is fabricated using 25% 6-carbon carboxylate SAMs, the gain improves to 188 ± 6% (Figure 3D), while the *K*_d_ is decreased until 23.6 ± 0.9 μM (Figure 3G). Further increases in the carboxylate content result in still further monotonic improvements in gain without any significant further change in *K*_d_. For example, the gain reached 474 ± 14% at 75% 6-carbon carboxylate, the highest 6-carbon carboxylate fraction we employed in our work. As a control, we verified the electrochemical inactivity of the 6-carbon carboxylate SAM on a gold electrode in PBS (i.e., without any surface-bound aptamer), thereby confirming that the improved EAB sensor response is due to the interaction between the carboxylate headgroup and the methylene-blue-modified aptamer (Figure S1). We observed similar improvements in the gain for our other two sensors. Specifically, the gain (at 100 μM target) of our vanc-28 sensor (Figure 3B) increased from 112 ± 1% for the carboxylate-free SAM to 183 ± 2% at a 6-carbon carboxylate fraction of 75% (Figure 3E), albeit with little change in *K*_d_ (Figure 3H). Finally, the gain (at 14 mM target) of our tryptophan sensor, which exhibits biphasic binding (Figure 3C),^38^ increased from 438 ± 7% to 2410 ± 163% as the 6-carbon carboxylate content rose from 0% to 75% (Figure 3F). Its *K*_d_, however, remained almost unchanged (Figure 3I). The improved gain seen for EAB sensors fabricated using mixed 6-carbon hydroxyl/6-carbon carboxylate SAMs translates into improved LOD (Figure 3A–C). Here, we arbitrarily defined the LOD as a KDM signal change of 0.3. For example, for 75% 6-carbon carboxylate, the LOD of the vanc-45 sensor is improved 3.2-fold relative to the 1.9 μM seen for the homogeneous 6-carbon hydroxyl. At 75% 6-carbon carboxylate, the LOD of the vanc-28 sensor improved 1.7-fold to 70 nM compared to the homogeneous 6-carbon hydroxyl SAM. Similarly, at 60% 6-carbon carboxylate, the LOD of the tryptophan sensor improves by 8.5-fold relative to 1.7 μM seen for the homogeneously 6-carbon hydroxyl-terminated surface.

**Figure 4:**
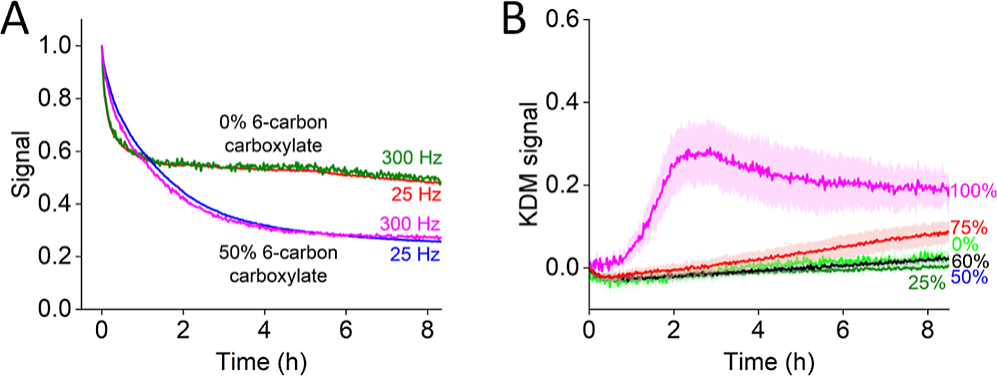
EAB sensors employing mixed carboxylate SAMs remain “drift correctable” when challenged in undiluted whole blood held at 37 °C. (A) Under these conditions, the signals produced by vanc-45 sensors employing a homogeneous hydroxyl-terminated SAM or 50% 6-carbon carboxylate SAM both decrease significantly over time. (B) Employing KDM, however, accurately corrects this drift for sensors employing 6-carbon carboxylate headgroups at levels as high as 60%.

In addition to being stable in buffer, sensors fabricated using mixed 6-carbon carboxylate- and 6-carbon hydroxyl-terminated SAMs are also drift-correctable when challenged in undiluted whole blood at physiological temperatures. Previously we have shown that, due to fouling and disruption of the SAM,^30^ the SWV signals produced at individual square-wave frequencies drift significantly for the homogeneously 6-carbon hydroxyl-terminated SAMs. Here, we find that this is also true for mixed 6-carbon carboxylate- and 6-carbon hydroxyl-terminated SAMs (Figure 4A), with the drift proving most significant for the 50% 6-carbon carboxylate surface. We presume this occurs because the negatively charged SAM promotes nonspecific electrostatic interactions with blood components.^43^ Fortunately, however, KDM drift correction works well for sensors employing 6-carbon carboxylate contents as great as 60% (Figure 4B).^36,37^ The drift associated with higher 6-carbon carboxylate contents, in contrast, is corrected rather poorly.

**Figure 5:**
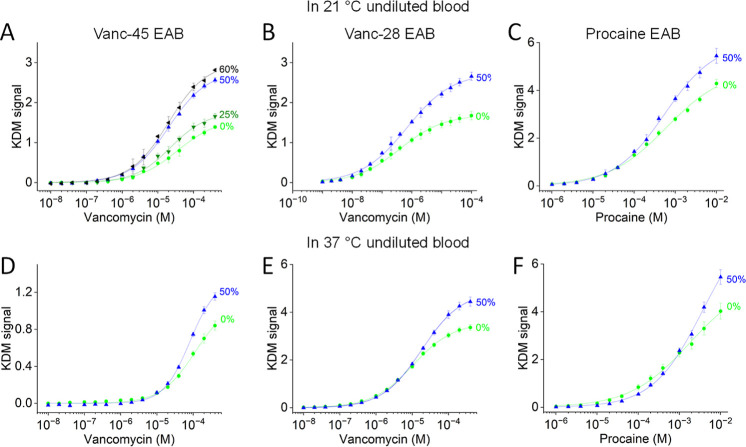
EAB sensor performance in undiluted blood is also improved upon the introduction of substoichiometric levels of 6-carbon carboxylate headgroups in the SAM. Shown are the responses of (A,D) vanc-45, (B,E) vanc-28, and (C,F) procaine sensors employing varying fractions of 6-carbon carboxylate in their SAM when they are challenged in undiluted blood at (A–C) room temperature or (C–F) physiological temperature.

The improved gain and LOD seen for mixed 6-carbon carboxylate- and 6-carbon hydroxyl-terminated SAMs in room-temperature PBS also hold when the sensors are deployed in room-temperature whole blood (Figure 5A–C and Table S2). Specifically, under these conditions, vanc-45 sensors employing a homogeneously 6-carbon hydroxyl-terminated SAM produce a gain of 139 ± 3% (at 400 μM target), a LOD of 5.3 μM, and a *K*_d_ of 35.1 ± 4.5 μM (Figure 5A). And the gain improved to 281 ± 4% and the LOD to 1.4 μM, respectively, at 60% 6-carbon carboxylate. We hypothesize that the reduced impact we see on gain and LOD in blood (relative to that in PBS) is due to fouling, which presumably reduces the distinctions between the carboxylate- and hydroxyl-terminated SAMs. Even the poorer LOD, however, is sufficient to monitor clinically relevant vancomycin concentrations in serum, which range between 6 and 35 μM.^44^ In contrast to LOD and gain, the aptamer *K*_d_ was not significantly affected by increasing carboxylate content, with a *K*_d_ of 19.8 ± 1.5 μM at 60% 6-carbon carboxylate. The gain (at 100 μM target) and LOD of the vanc-28 sensor similarly improved, rising from 168 ± 11% and 30 nM for the hydroxyl-terminated surface to 266 ± 10% and 20 nM, respectively, at 50% carboxylate (Figure 5B). The *K*_d_ changed from 400 ± 40 nM for the homogeneously 6-carbon hydroxyl-terminated SAM to 680 ± 80 nM for the 50% 6-carbon carboxylate SAM.

As the presence of endogenous tryptophan in blood complicates the characterization of the tryptophan sensor, we excluded this sensor from our tests in blood. Instead, to further explore the generality of our observations, we added to our testing a previously described procaine-detecting EAB sensor^23^ (Figure 5C). The gain (at 10 mM target) of this sensor improved, shifting from 429 ± 17% for the hydroxyl-terminated surface to 544 ± 31% for the 50% 6-carbon carboxylate surface when challenged in room-temperature whole blood. Its LOD, in contrast, remained almost unchanged (7.3 versus 8.6 μM). Of note, this LOD is sufficient to detect procaine at clinically relevant concentrations (40–180 μM).^45^ We were unable to ascertain whether this sensor’s *K*_d_ changed upon changing the monolayer surface, as we could not reach the upper plateau in the calibration curve.

The improved gain upon the introduction of substoichiometric levels of 6-carbon carboxylate in the SAM holds also in blood at physiological temperatures, although less significantly under these conditions (Figure 5D–F and Table S2). The gain (at 400 μM target) of the vanc-45 sensor in 37 °C whole blood increases from 84 ± 5% to 116 ± 4% as the 6-carbon carboxylate content increases from 0 to 50%, which is insufficient to significantly alter the LOD (Figure 5D). Unfortunately, under these conditions, we were unable to determine the *K*_d_ of this sensor, as we could not reach experimentally the upper plateau in the calibration curve. Under the same conditions, the gain (at 400 μM target) of the vanc-28 sensor improved from 336 ± 12% for the hydroxyl-terminated surface to 444 ± 19% for a 50% 6-carbon carboxylate surface, with the LOD again remaining unchanged and the *K*_d_ changing only from 10.4 ± 0.3 μM to 18.3 ± 0.5 μM (Figure 5E). The decrease in affinity compared to the same sensor employed in blood at 21 °C has been observed before.^46^ Finally, the gain of the procaine sensor (at 10 mM target) improved from 402 ± 33% to 545 ± 31% as the 6-carbon carboxylate content was increased from 0 to 50%, with the LOD simultaneously falling from 1.6 to 3.9 μM (Figure 5F).

**Figure 6:**
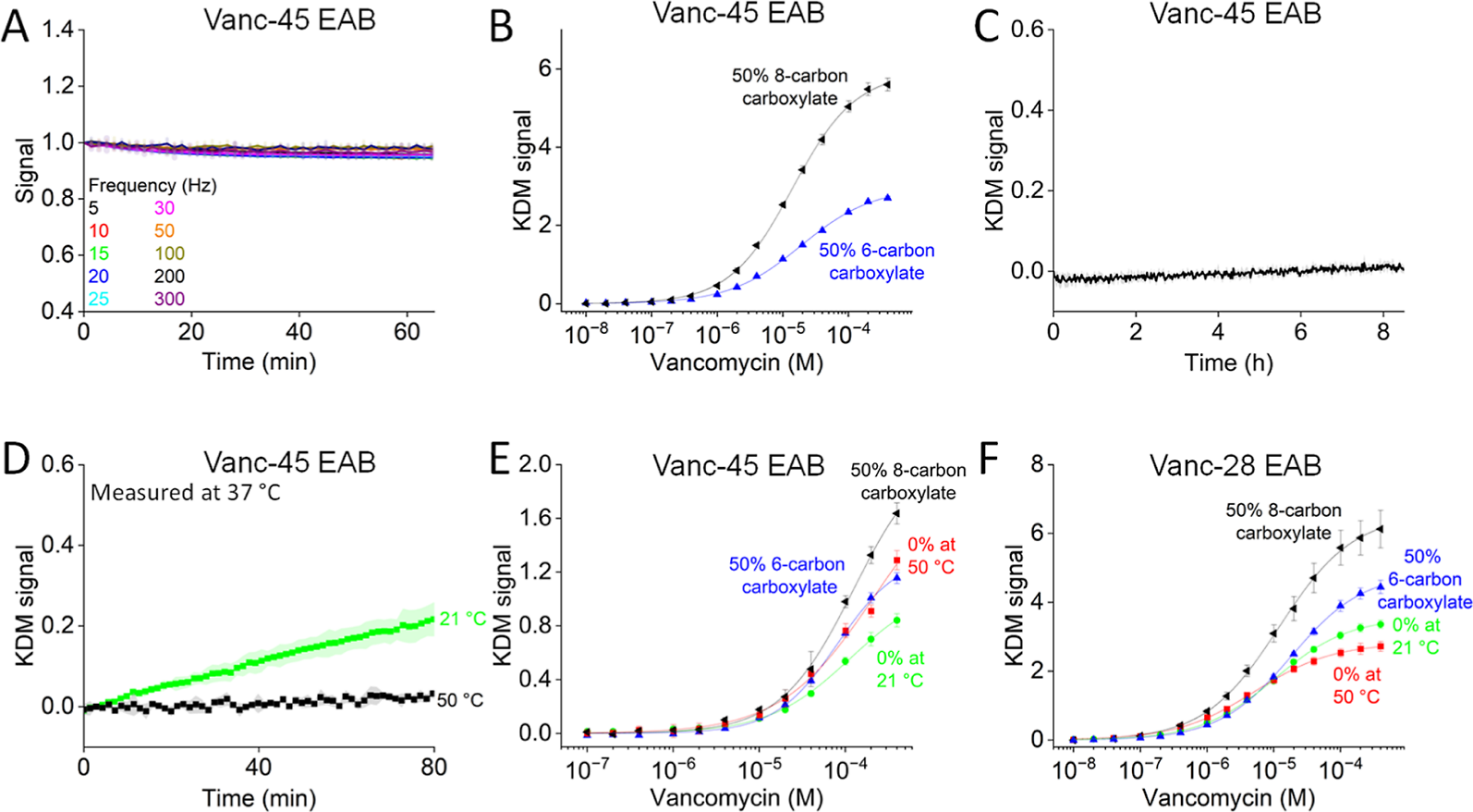
Use of mixed, 6-carbon hydroxyl, and 8-carbon carboxylate SAMs leads to still greater improvement in sensors gain both in room-temperature buffer and undiluted blood at room temperature and 37 °C. (A) Signal and (B) titration data of vanc-45 with 50% 8-carbon carboxylate in PBS at room temperature. Displayed in (B) is also the titration data of vanc-45 with 50% 6-carbon carboxylate. (C) KDM signal of vanc-45 with 50% 8-carbon carboxylate in undiluted blood at 37 °C. (D) KDM signal of vanc-45 with 50% 8-carbon carboxylate in PBS at 37 °C. We formed the SAM at 21 or 50 °C. Titration data of (E) vanc-45 and (F) vanc-28 sensors with 50% 8-carbon carboxylate in undiluted blood at 37 °C. Also shown are titrations of vanc-45 and vanc-28 with 0% carboxylate with a SAM formed at room temperature or 50 °C and 50% 6-carbon carboxylate.

The use of a mixed SAM with 8-carbon carboxylate and 6-carbon-hydroxyl improves EAB sensor performance even more than mixed SAMs employing 6-carbon carboxylate thiols. Our studying this stemmed from our hypothesis that increasing the spacer length from 6 to 8 carbons would place the carboxylate closer to the aptamer, thus increasing the electrostatic repulsion between the carboxylate and aptamer and potentially further improving performance. To test this, we fabricated vanc-45 sensors employing a SAM containing 50% 8-carbon carboxylate and 50% 6-carbon hydroxyl. By challenging these in PBS at room temperature, we obtained a stable signal (Figure 6A). Under these conditions, the gain (at 400 μM target) of the vanc-45 sensor improved from 270 ± 5% to 560 ± 16%, the *K*_d_ improved from 18.8 ± 0.9 μM to 14.1 ± 0.4 μM, and the LOD improved from 1.2 μM to 0.6 μM relative to sensors fabricated using 50% 6-carbon carboxylate (Figure 6B and Table S3).

Sensors fabricated from 50% 8-carbon carboxylate achieve good gain and remain drift-correctable when challenged in whole blood at 37 °C (Figure 6C). This said, to achieve a stable KDM signal with this SAM required that we perform the SAM deposition at 50 °C rather than, as previously employed, room temperature (Figure 6D, elevated fabrication temperatures improve SAM homogeneity^47,48^). The gain (at 400 μM target) of the resulting vanc-45 sensor is 164 ± 8%, representing a significant improvement relative to the 84 ± 5%, 129 ± 7%, and 116 ± 4% gains we obtained from a homogeneous 6-carbon hydroxyl SAM fabricated at room temperature or 50 °C or a 50% 6-carbon carboxylate SAM, respectively (Figure 6E and Table S4). We hypothesize that the formation of the SAM at 50 °C reduces the adherence of unfolded aptamers to the electrode surface which, by reducing the number of target-unresponsive oligonucleotides, would increase gain.^49^ We did not, however, observe any significant improvement in the LOD.

We observed a similar improvement in gain for the vanc-28 sensor upon usage of 50% 8-carbon carboxylate (Figure 6F and Table S4). Its gain (at 400 μM target) equals 613 ± 55%, which is an improvement compared to the gains of 336 ± 12%, 272 ± 14%, and 444 ± 19% from a homogeneous 6-carbon hydroxyl SAM fabricated at room temperature or 50 °C and a 50% 6-carbon carboxylate SAM, respectively. Similarly, the LOD improved from 490 nM to 240 nM upon changing from a 6-carbon hydroxyl-terminated surface formed at room temperature to a 50% 8-carbon-carboxylate formed at 50 °C. Conversely, upon the same change, the *K*_d_ only shifted from 10.4 ± 0.3 μM to 12.0 ± 0.5 μM.

**Figure 7:**
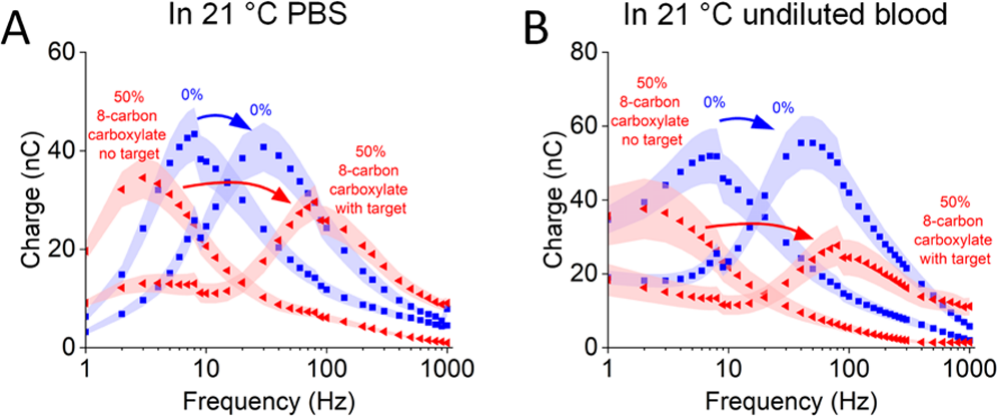
Greater gain associated with a 50% 8-carbon carboxylate SAM arises due to deceleration of electron transfer from the unbound aptamer and acceleration of transfer from the bound aptamer. To illustrate this, here we present Lovrić plots^50^ (charge transfer versus square-wave frequency) for vanc-45 sensors in the unbound and bound state (400 μM vancomycin) in (A) PBS and (B) undiluted blood, both at room temperature. Here, we formed the 50% 8-carbon carboxylate SAM at 50 °C and the 0% carboxylate SAM at room temperature.

EAB sensor gain is defined by the difference in the electron transfer rates associated with the unfolded, unbound aptamer and the folded, target-bound aptamer (Figure 1A). Consistent with this, the switch from a homogeneously hydroxyl-terminated, 6-carbon surface to a 50% 8-carbon carboxylate SAM slows the rate of electron transfer from the unbound vanc-45 by a factor of 2.6 in PBS and 4 in whole blood, as determined using Lovrić plots (Figure 7).^50^ Similarly, the electron transfer rates seen for the target-bound state increase by factors of 2.6 and 2 in the buffer and blood, respectively. We presume the former occurs because the rate of electron transfer from a methylene blue at the distal end of a single-stranded, electrode-bound oligonucleotide is limited by the rate with which this reporter diffuses to the electrode surface,^51^ a mechanism that would be slowed by the increased negative charge on the carboxylate-modified surface.^52^ The accelerated transfer of the folded state, in contrast, is presumably caused by the stronger interaction between the negatively charged SAM and the positively charged methylene blue, thus holding the methylene blue more rigidly to the surface.

## Conclusions

Mixed monolayers composed of 6-carbon hydroxyl-terminated thiols and varying amounts of 6- or 8-carbon, carboxylate-terminated thiols are advantageous to use as they can significantly improve the gain of EAB sensors at both room temperature and physiological temperature in both simple, buffered solutions and in undiluted whole blood. Employing the carboxylate-based sensor does not affect the stability of EAB sensors in whole blood, making this approach applicable for both ex vivo point-of-care diagnostics and in vivo real-time applications.

## Experimental Section

### Materials

A gold wire with a diameter of 0.2 mm and l-tryptophan were purchased from Thermo Fisher Scientific. Polyolefin heat-shrink tubing was purchased from McMaster-Carr (Santa Fe Springs, California). PBS was obtained from ChemCruz. Magnesium chloride hexahydrate was obtained from Fisher Scientific. Aptamers modified with a thiol at the 5′ end and methylene blue at the 3′ were obtained from Integrated DNA Technologies (Coralville, Iowa). The relevant aptamer sequences are given in Table 1. 6-Mercaptohexanol, 6-mercaptohexanoic acid, 8-mercaptooctanoic acid, tris(2-carboxyethyl)phosphine hydrochloride (TCEP), and sodium hydroxide were purchased from Sigma-Aldrich. Vancomycin hydrochloride and sulfuric acid were obtained from VWR. Bovine blood in heparin was purchased from HemoStat laboratories (Dixon, California). The Ag/AgCl reference electrode and platinum wire counter electrode were obtained from CH instruments (Austin, Texas).

**Table 1:** Aptamer Sequences Used in This Study

vanc-28	5′-HS-GCG AGG GTA CCG CTT AAA GTG GGT CGG C-methylene blue-3′
vanc-45	5′-HS-CGA GGG TAC CGC AAT AGT ACT TAT TGT TCG CCT ATT GTG GGT CGG-methylene blue-3′
tryptophan	5′-HS-CCG GTG GTG TAG TTC CGG CGT GGG GAA GG-methylene blue-3′
procaine	5′-HS-AGA CAA GGA AAA TCC TTC AAC GAA GTG GGT CG-methylene blue-3′

The sequences of vanc-45, tryptophan, and procaine were adopted from the literature.^18,23,38^ The sequence of vanc-28 was kindly provided by Professor M. Stojanovic of Columbia University, who will describe its isolation in a separate publication.

## Methods

### Sensor Fabrication

EAB sensors were fabricated using standard protocols.^38,39,46^ In brief, a 4.5 cm gold wire was soldered to an electrochemical connector for connection to the potentiostat and insulated by using 3.9 cm of heat shrinkable polyolefin tubing. The uncovered gold wire was cut to a length of 3 mm. To clean the gold, the wire was immersed first in a 0.5 M NaOH solution and subjected to electrochemical cleaning using a potential window from −1.0 V to −2.0 V (all potentials versus Ag/AgCl) at a scan rate of 1 V/s for 1000 cycles using a CH1040C potentiostat (CH Instruments) in a three-electrode setup using a platinum counter electrode and a Ag/AgCl reference electrode. Next, the microscopic roughness of the gold wire was increased by placing the wire in a 0.5 M H_2_SO_4_ solution and pulsed from 0.0 to 2.2 V using a pulse width of 0.02 s, which was repeated for 32,000 cycles. The degree of surface roughening was verified by determination of the electrode surface area in a 0.5 M H_2_SO_4_ solution using a potential window from 0 to 1.8 V at a scan rate of 1 V/s for 10 cycles.

We fabricated sensors from clean gold wires as follows. The relevant aptamer was dissolved in water, aliquoted, and stored at −20 °C until use. Immediately prior to use, the aptamer was reduced for 1 h in a solution containing 11.26 μM aptamer and 8.87 mM TCEP and subsequently diluted until 500 nM in PBS plus 2 mM MgCl_2_. The cleaned gold wire was immersed in the reduced aptamer solution for 1 h. Afterward was the gold wire rinsed with Milli-Q water.

For SAMs consisting of 6-mercaptohexanol and or 6-mercaptohexanoic acid, the gold wire was then immersed overnight in a solution containing 6-mercaptohexanol and or 6-mercaptohexanoic acid at the desired stoichiometric ratio up to a carboxylate content of 75% and a total thiol concentration of 10 mM. For the formation of a 100% 6-carbon carboxylate SAM, the electrode was immersed for 1 h in 10 mM 6-mercaptohexanoic acid, washed with Milli-Q water, and immersed in 10 mM 6-mercaptohexanol overnight. For SAMs consisting of 8-mercaptooctanoic acid, the gold wire was then immersed overnight in a solution consisting of 50% 6-mercaptohexanol and 50% 8-mercaptooctanoic acid. The SAM was formed at room temperature and 50 °C for experiments conducted at room temperature and 37 °C, respectively. The sensor was then washed with Milli-Q water prior to use.

## Electrochemical Measurements

The electrochemical response of the EAB sensor was measured with SWV using a potential window of −0.15 V to −0.45 V, an amplitude of 0.025 V, and frequencies between 1 and 1000 Hz. The electrochemical response during titration experiments was measured after an equilibration time of 3 min following target addition. The sensor response was monitored continuously during the stability measurements. The data was normalized and subjected to KDM analysis.^36^ For SAMs consisting of 6-mercaptohexanol and/or 6-mercaptohexanoic acid: for the vanc-48 sensor, we used frequency pairs of 10 and 300 Hz and 25 and 300 Hz in experiments conducted at room temperature and 37 °C, respectively. For vanc-28, we used 10 and 200 Hz under all conditions, while for both the tryptophan and procaine sensors, we used 10 and 300 Hz. For SAMs consisting of 8-mercaptooctanoic acid: for the vanc-48 sensor, we used frequency pairs of 10 and 300 Hz and 25 and 200 Hz in experiments conducted at room temperature and 37 °C, respectively. For the vanc-28 sensor, we used 10 and 200 Hz. Lovrić plots were measured between 1 and 1000 Hz.

## Supporting Information Available

Tables summarizing the *K*_d_, gain, and LOD of various EAB sensors and square-wave voltammograms (PDF)
