# Supplementary material for: Carboxylate-Terminated Electrode Surfaces Improve the Performance of Electrochemical Aptamer-Based Sensors
Source: ACS Appl Mater Interfaces. 2025 Jan 22;17(5):8706–14. doi: 10.1021/acsami.4c21790 (PMC11803614; doi:10.1021/acsami.4c21790)
Supplement: Supplementary file 1 — am4c21790_si_001.pdf [file am4c21790_si_001.pdf]

**Supporting information**

# Carboxylate-terminated electrode surfaces improve the performance of electrochemical aptamer-based sensors

Rose Mery Bakestani[1], Yuyang Wu[1], Bettina Glahn-Martínez[1,2], Tod E. Kippin[3], Kevin W. Plaxco[1,4], and Ruben W. Kolkman[1,*]

[1]Department of Chemistry and Biochemistry, University of California Santa Barbara, Santa Barbara, CA 93106, USA

[2]Department of Analytical Chemistry, Faculty of Chemistry, Universidad Complutense de Madrid, 28040 Madrid, Spain

[3]Department of Psychological and Brain Sciences, University of California Santa Barbara, Santa Barbara, California 93106, USA

[4]Biological Engineering Graduate Program, University of California Santa Barbara, Santa Barbara, CA 93106, USA

*Corresponding author: kolkman@ucsb.edu

**Table S1.** $K_d$, gain, and LOD of the vanc-45 EAB, vanc-28 EAB, and tryptophan EAB sensors using varying fractions of 6-carbon carboxylate-terminated thiols in the SAM. The sensors were tested in PBS buffer at room temperature. Here we defined signal gain as the relative signal change between 0 and 400 µM vancomycin (vanc-48), 0 and 100 µM vancomycin (vanc-28), and between 0 and 14 mM tryptophan.

| | 6-carbon carboxylate in SAM (%) | $K_d$ (µM) | Signal gain (%) | LOD (µM) |
|---|---|---|---|---|
| Vanc-45 EAB | 0% | 15.2 ± 0.4 | 173 ± 2 | 1.9 |
| | 25% | 23.6 ± 0.9 | 188 ± 6 | 2.4 |
| | 50% | 18.9 ± 0.9 | 297 ± 5 | 1.2 |
| | 60% | 17.7 ± 0.8 | 368 ± 10 | 0.8 |
| | 75% | 16.6 ± 0.7 | 474 ± 14 | 0.6 |
| Vanc-28 EAB | 0% | 0.6 ± 0.1 | 112 ± 1 | 0.12 |
| | 25% | 1.0 ± 0.1 | 134 ± 7 | 0.13 |
| | 50% | 1.2 ± 0.2 | 170 ± 2 | 0.08 |
| | 60% | 1.1 ± 0.1 | 174 ± 15 | 0.07 |
| | 75% | 1.0 ± 0.1 | 183 ± 2 | 0.07 |
| Tryptophan EAB | 0% | 13.0 ± 1.4 | 438 ± 7 | 1.7 |
| | 25% | 7.0 ± 0.5 | 787 ± 16 | 0.4 |
| | 50% | 9.1 ± 0.4 | 1413 ± 113 | 0.3 |
| | 60% | 7.2 ± 0.2 | 1509 ± 105 | 0.2 |
| | 75% | 11.1 ± 0.4 | 2410 ± 163 | 0.2 |

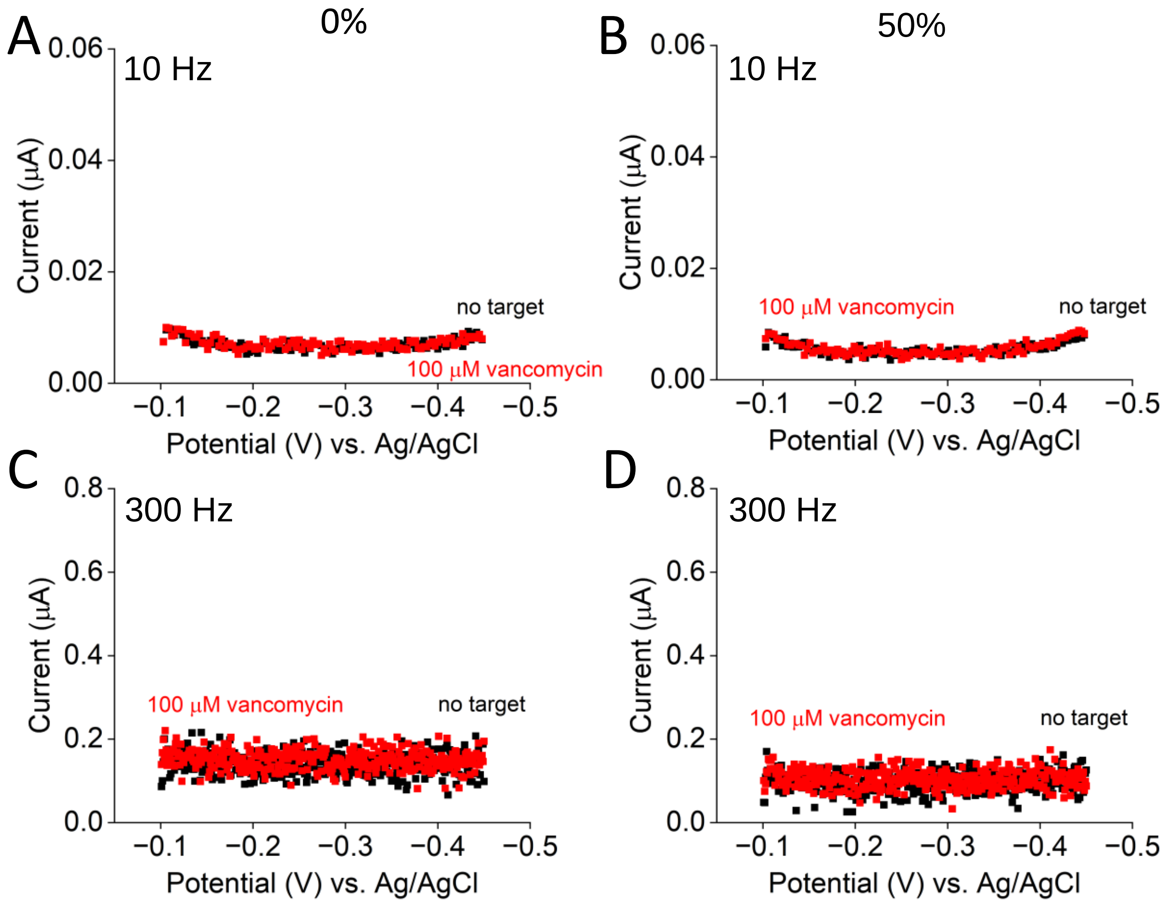


**Figure S1.** Representative square wave voltammogram of a gold electrode modified with a thiol-based SAM with **(A, C)** 0% and **(B, D)** 50% 6-carbon carboxylate, measured at **(A, B)** 10 Hz and **(C, D)** 300 Hz. The voltammograms were measured in PBS buffer at room temperature, with or without 100 μM vancomycin. Note that no aptamer was deposited in these experiments.

**Table S2.** $K_d$, gain, and LOD of the vanc-45 EAB and vanc-28 EAB sensors using varying fractions of 6-carbon carboxylate-terminated thiols in the SAM. The sensors were tested in undiluted blood at room temperature and 37 °C. Here we defined signal gain as the relative signal change between 0 and 400 µM vancomycin (vanc-48 and at room temperature for vanc-28) and 0 and 100 µM vancomycin (vanc-28 at 37 °C).

| | 6-carbon carboxylate in SAM (%) | $K_d$ (µM) | | Signal gain (%) | | LOD (µM) | |
|---|---|---|---|---|---|---|---|
| | | 21 °C | 37 °C | 21 °C | 37 °C | 21 °C | 37 °C |
| Vanc-45 EAB | 0% | 35.1 ± 4.5 | N.A. | 139 ± 3 | 84 ± 5 | 5.3 | 38.4 |
| | 25% | 22.8 ± 2.9 | N.A. | 166 ± 7 | N.A. | 3.6 | N.A. |
| | 50% | 20.2 ± 1.4 | N.A. | 256 ± 6 | 116 ± 4 | 1.5 | 28.8 |
| | 60% | 19.8 ± 1.5 | N.A. | 281 ± 4 | N.A. | 1.4 | N.A. |
| vanc-28 EAB | 0% | 0.40 ± 0.04 | 10.4 ± 0.3 | 168 ± 11 | 336 ± 12 | 0.03 | 0.49 |
| | 50% | 0.68 ± 0.08 | 18.3 ± 0.5 | 266 ± 10 | 444 ± 19 | 0.02 | 0.62 |
| Procaine EAB | 0% | N.A. | N.A. | 429 ± 17 | 402 ± 33 | 7.3 | 1.6 |
| | 50% | N.A. | N.A. | 544 ± 31 | 545 ± 31 | 8.6 | 3.9 |

**Table S3.** $K_d$, gain, and LOD of the vanc-45 EAB sensor using varying fractions of 6-carbon carboxylate or 8-carbon carboxylate-terminated thiols in the SAM. The sensors were tested in PBS buffer at room temperature. Here we defined signal gain as the relative signal change between 0 and 400 µM vancomycin.

| | 6-carbon carboxylate in SAM (%) | 8-carbon carboxylate in SAM (%) | $K_d$ (µM) | Signal gain (%) | LOD (µM) |
|---|---|---|---|---|---|
| Vanc-45 EAB | 50% | | 18.8 ± 0.9 | 270 ± 5 | 1.2 |
| | | 50% | 14.1 ± 0.4 | 560 ± 16 | 0.6 |

**Table S4.** $K_d$, gain, and LOD of the vanc-45 EAB and vanc-28 EAB sensors using varying fractions of 6-carbon carboxylate or 8-carbon carboxylate-terminated thiols in the SAM. The sensors were tested in undiluted blood at 37 °C. Here we defined signal gain as the relative signal change between 0 and 400 μM vancomycin (vanc-48 and vanc-28).

| | 6-carbon carboxylate in SAM (%) | 8-carbon carboxylate in SAM (%) | $K_d$ (μM) | Signal gain (%) | LOD (μM) |
|---|---|---|---|---|---|
| Vanc-45 EAB | 0% (formed at RT) | | N.A. | 84 ± 5 | 38.4 |
| | 0% (formed at 50 °C) | | N.A. | 129 ± 7 | 24.2 |
| | 50% | | N.A. | 116 ± 4 | 28.8 |
| | | 50% | N.A. | 164 ± 8 | 21.0 |
| Vanc-28 EAB | 0% (formed at RT) | | 10.4 ± 0.3 | 336 ± 12 | 0.49 |
| | 0% (formed at 50 °C) | | 5.2 ± 0.2 | 272 ± 14 | 0.30 |
| | 50% | | 18.3 ± 0.5 | 444 ± 19 | 0.62 |
| | | 50% | 12.0 ± 0.5 | 613 ± 55 | 0.24 |
